# Structure-Guided Design of Partial Agonists at an Opioid Receptor

**DOI:** 10.21203/rs.3.rs-4664764/v1

**Published:** 2024-07-16

**Authors:** Tao Che, Balazs Varga, Sarah M Bernhard, Amal El Daibani, Saheem Zaidi, Jordy Lam, Jhoan Aguilar, Kevin Appourchaux, Antonina Nazarova, Alexa Kouvelis, Shainnel Eans, Elyssa Margolis, Jonathan Fay, Amynah Pradhan, Vsevolod Katritch, Jay McLaughlin, Susruta Majumdar

**Affiliations:** Washington University in St. Louis; WUSTL; Washington University Medical Center; Washington University; University of Southern California; University of Southern California; Washington University in St. Louis; Washington University in St. Louis; University of Southern California; Washington University; University of Florida; University of California, San Francisco; University of Maryland; Center for Clinical Pharmacology; USC; University of Florida; Washington University School of Medicine

## Abstract

The persistence of chronic pain and continuing overdose deaths from pain-relieving opioids targeting μ opioid receptor (μOR) have fueled the need for reliable long-term analgesics which use different targets and mechanisms. The δ opioid receptor (δOR) is a potential alternative target for non-addictive analgesics to alleviate chronic pain, made more attractive by its lack of respiratory depression associated with μOR agonists. However, early δOR full agonists were found to induce seizures, precluding clinical use. Partial δOR agonists may offer more controlled activation of the receptor compared to full agonists, but the development of such ligands has been hindered by uncertainty over the molecular mechanism mediating partial agonism. Using a structure-based approach, we explored the engagement of the sodium binding pocket in δOR and developed a bitopic ligand, C6-Quino, predicted to be a selective δOR partial agonist. Functional studies of C6-Quino revealed that it displayed δOR partial agonist activity at both G-protein and arrestin pathways. Its interaction with the sodium pocket was confirmed and analyzed using a single particle cryo-EM. Additionally, C6-Quino demonstrated favorable chemical and physiological properties like oral activity, and analgesic activity in multiple chronic pain models. Notably, μOR-related hyperlocomotion and respiratory depression, and δOR-related convulsions, were not observed at analgesic doses of C6-Quino. This fundamentally new approach to designing δOR ligands provides a blueprint for the development of partial agonists as safe analgesics and acts as a generic method to optimize signaling profiles of other Class A GPCRs.

## INTRODUCTION

The last two decades have witnessed the persistence of pain in all segments of the US population and a drastic increase in the consequences of adverse effects of opioids. Opioid based pain management has targeted μ opioid receptor (μOR) agonism, a highly effective approach in the treatment of acute pain, but the overuse of μOR agonists in both clinical and illicit use has led to a major public health crisis due to their severely aversive side effects, including impaired GI transit, antinociceptive tolerance, potential for abuse and addiction, and a potentially lethal respiratory depression^1^. Alternative strategies employed to safely harness the potential of diverse (κ opioid receptor (κOR), δ opioid receptor (δOR) and μOR) human opioid systems remain elusive, despite their great appeal. Studies of the δ-opioid receptor (δOR) identify three unique features recommending it as an ideal target for pain management: 1) The expression level of δORs is upregulated in chronic pain states^2^; 2) δOR agonists are devoid of most of the negative side effects associated with μOR agonists^3^; and 3) δOR agonists are effective against headache disorders and migraine^4^. Unfortunately, first generation δOR agonists like BW373U86, SNC80, and SNC162 exhibited anti-hyperalgesic properties but also caused convulsions at higher doses^5^. Later generation molecules, including ARM390, TAN67, and ADL5859, showed no seizure-inducing effects but had other limitations. Like other δOR agonists, ARM390 developed analgesic tolerance following chronic administration although it displayed low internalization capability at δOR^6^. (±)-TAN67’s effect was unpredictable due to polypharmacology at other targets (e.g., MRGPRX2)^7^ and unexpected off-target effects^8–10^. ADL5859 did not meet primary endpoints in phase 2 clinical trials targeting osteoarthritis in the knee^11^. Two recently developed G protein-biased δOR agonists, TRV250 and PN6047, have both completed a phase I clinical trial for neuropathic pain^12,13^. The correlations between G-protein and arrestin signaling with *in vivo* pharmacology at δOR has not been fully elucidated. It appears that adverse effects are multifactorial, possibly caused by the activation of specific G protein subtypes ^14,15^ in addition to activation of GRK subtypes and distinct G_βγ_ subunits^16^.

Emerging evidence suggests that reduced intrinsic efficacy for G protein activation could lead to improved side effect profiles for opioids^17–20^, antipsychotics^21,22^ and non-hallucinogenic psychedelic analogs^23^. This evidence highlights the potential opportunity for pursuing δOR partial agonists as therapeutic agents with improved safety profiles. Partial agonists produce a submaximal response compared to full agonists and have been associated with fewer side effects including opioid physical dependence. Unfortunately, the molecular mechanism mediating partial agonism remains unclear, severely hindering the rational design of such ligands. What is the key mechanism of partial agonism and what is its structural basis, remain key questions to be resolved.

Recently, structural advances focusing on the conserved sodium site in a highly diverse array of class A GPCRs have proposed this site as an “efficacy-switch” controlling ligand efficacy^24–28^. Sodium acts as a negative allosteric modulator^29^, and is critical for the control of signaling in a number of GPCRs at physiological concentrations. Specifically, the sodium pocket is shown to undergo dramatic conformational changes upon receptor activation, with recent studies proving residues in the sodium pocket control the basal activity of the receptor and differentially modulate GPCR activation towards signaling at either G-protein or β-arrestin pathways^30–34^. Consistent with this, the high-resolution inactive state structure of δOR revealed a sodium ion in a pocket at the bottom of the orthosteric site^35^. Several mutations in the δOR sodium pocket converted the action of the δOR antagonist, naltrindole (NTI), into partial or full agonism in the βarrestin-2 pathway, further validating the sodium site as an attractive target to control ligand efficacy and modulate signaling activity through the δOR.

**Figure 1:**
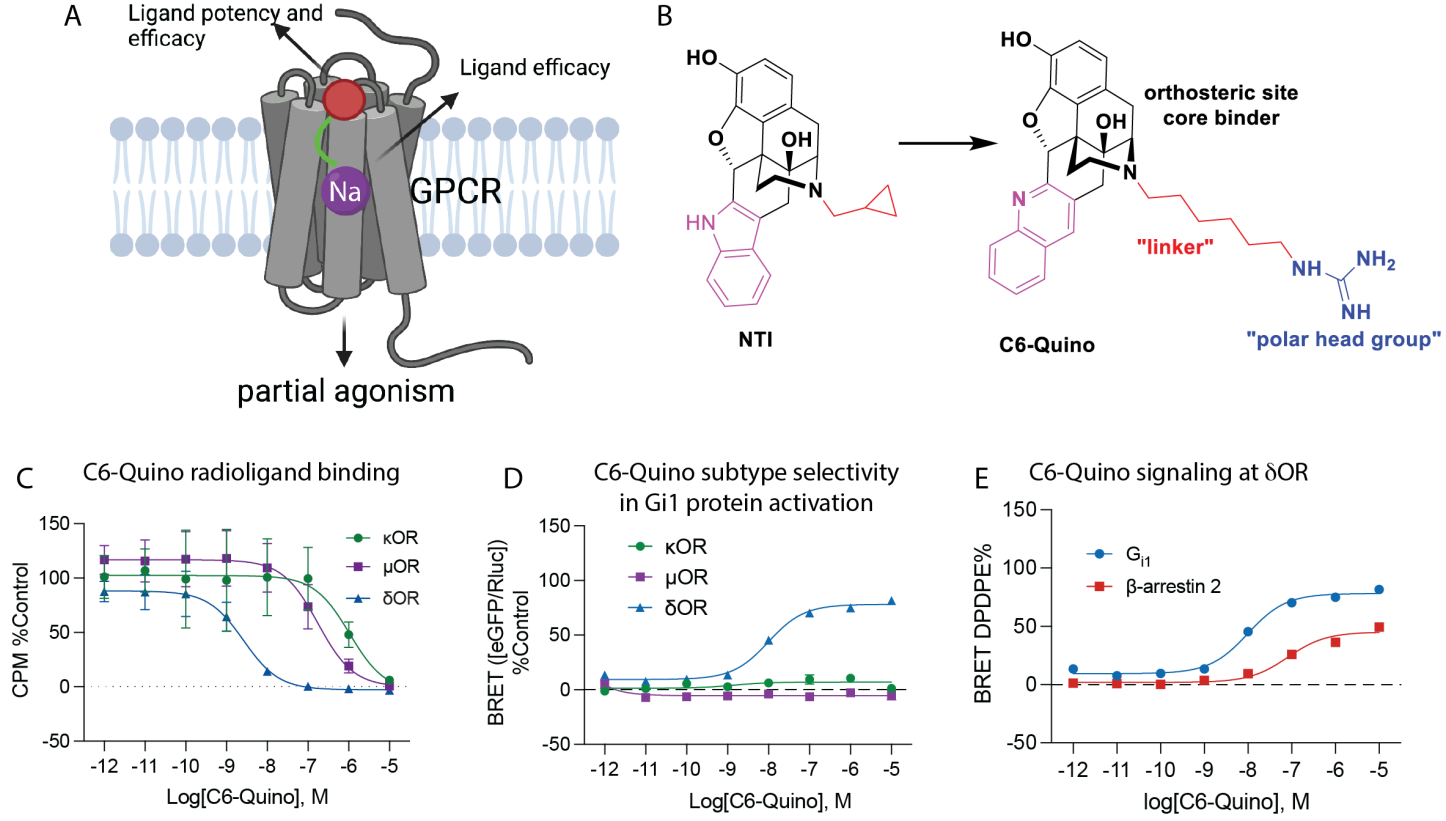
Strategy for design of δOR partial agonist. A) Ligand efficacy can be modulated through the allosteric site, while the orthosteric site controls potency and efficacy. B) Design of δOR partial agonist C6-Quino. C) Binding, D) functional selectivity of C6-Quino at μOR, κOR, δOR and E) Gi1 and β-arrestin 2 signaling of C6-Quino at δOR, referenced to DPDPE. Potency and efficacy values for panels C, D and E are shown in Tables S1, S2 and S3.

In the present study, we used a structure-based approach and rationally designed a highly selective δOR partial agonist (**C6-Quino**) based on NTI, intended to function as a bitopic ligand by targeting both the orthosteric site and, with a polar head group, the sodium site (Fig. 1A). A transition from partial to full agonism was observed with a shorter length of the carbon-chain linker between the groups targeting the orthosteric core and the polar head group (C5-Quino). In both cell lines transfected with human δOR and rat whole cell electrophysiological recording from neurons in ventral tegmental area (VTA), **C6-Quino** displayed partial agonist activity compared to the full agonist **C5-Quino** and other typical δOR agonists. We then obtained single particle cryo-EM structures of **C5-Quino** (2.6 Å) and **C6-Quino** (2.8 Å) bound to δOR and confirmed their interaction with the sodium site. The cryo-EM structures coupled with molecular dynamics simulations revealed water-mediated interactions between the ligand functional groups and key residues in the sodium site which control efficacy at both G-protein and β-arrestin signaling pathways. In mice, **C6-Quino** exhibited antinociceptive activity in chronic pain models of neuropathic pain, inflammatory pain, and migraine. Unlike many existing δOR agonists, **C6-Quino** does not cause convulsions. **C6-Quino** also shows reduced hyperlocomotor activity and reduced respiratory depression compared to morphine. Together these properties suggest the utility of developing pharmacological bitopic entities for the treatment of chronic pain with limited undesired adverse effects.

## RESULTS

### Develop selective δOR bitopic ligands by targeting the orthosteric site

To achieve selective activation of the human delta opioid receptor (δOR), we explored the design of agonists starting with the indole structure adapted from naltrindole (NTI), a known selective δOR antagonist. For modeling, we used the NTI-bound high resolution inactive-state δOR X-ray crystal structure (PDB: 4N6H) with the sodium ion in a highly conserved and functionally critical sub-pocket^35^. This structure shows the distance between the basic amine of NTI and the carboxy group of D95^2.50^ (residues numbered according to Ballesteros-Weinstein numbering) residue of the allosteric sodium-binding site to be 11.2 Å. To engage the allosteric sodium binding site, we swapped the cyclopropylmethyl group of the NTI core (Fig. 1B) starting from the basic nitrogen using an aliphatic chain linker (C_n_ where n = 3, 5, 6 and 7) connected to a positively charged guanidine group (C3- to C7-guano) as a functional “warhead”.

We first identified two indole derivatives, named C5-Indole and C6-Indole as possessing the optimal linker lengths (5 carbon and 6 carbons, respectively, see Figures S1 through S6 for structures and synthesis of all analogs and Figure S4 in particular for indole core structures) to engage the sodium binding pocket. We examined G_i1_ signaling for these derivatives at κOR and μOR and found that C5-Indole showed approximately 50-fold selectivity for δOR over κOR while C6-Indole showed about 90-fold selectivity (Figure S7A and S7B). Neither of the two compounds showed agonist activity at μOR (Table S1). However, since C6-Indole was partially effective at κOR, maintaining 35% efficacy (Figure S7B), we decided to modify the chemical structure from indole to quinoline (see Figure S5 for structures and synthesis), aiming to increase δOR selectivity over κOR. In our δOR computational model, the hydrophobic indole moiety is nested in a very hydrophobic pocket lined by V^6.55^, W^6.58^, and L^7.35^ residues (Figure S7C). Slightly increasing the bulk of the ring by changing the indole to quinoline leads to steric clashes with the less flexible W^7.35^ in μOR and Y^7.35^ in κOR. In comparison, the more flexible L^7.35^ residue of δOR was able to accommodate this localized increase in ligand size (Figure S7C). Confirming this prediction, **C6-Quino** maintained high potency for δOR in signaling assays (Figure S7D) but did not show measurable signaling at κOR (Figure S7E) or μOR (Table S1). δOR subtype selectivity was further confirmed using affinity binding assays (Fig. 1C, Figure S8, Table S2). In summary, improved subtype selectivity in both functional and binding affinity assays (Fig. 1C, 1D, 1E, Figure S8, Table S2) was attained through the indole-quinoline modifications in the orthosteric site.

**C6-Quino** was screened across a ~ 317 target panel in the PRESTO TANGO assays using β-arrestin2 as the read out through the Psychoactive Drug Screening Program at the National Institute of Mental Health^36^. In this platform, the signal increased > 3-fold above basal levels only at cholinergic receptor muscarinic 5 (CHRM5). However, when a dose response analysis was carried out for CHRM5, **C6-Quino** displayed no agonism at this target, strongly suggesting this result to be a false positive (Figure S9).

### Efficacy modulation of δOR ligands by targeting the allosteric sodium site

**Figure 2:**
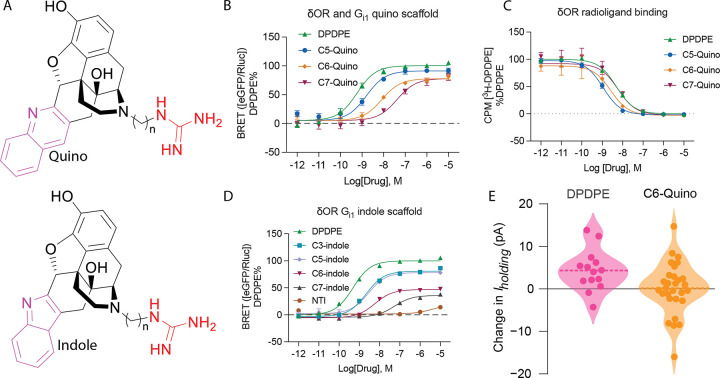
Profiling the signaling of C5, C6, and C7 analogs. (A) General structures of Quino and Indole scaffolds. (B) Gai-1 signaling of C5, C6, and C7 quino analogs at δOR using TRUPATH BRET assays. Ligand efficacy can be modulated through the allosteric site and is dependent on linker. Potency and efficacy values are shown in Table S3. (C) Radioligand binding of Quino compounds with different linker lengths. Figures contain data ± SD grouped from three independent biological replicates. Quantification of data can be found in Table S4. (D) Gai-1 signaling of C3, C5, C6, C7 indole analogs, and NTI at δOR using TRUPATH Gαβγ biosensors. Potency and efficacy values are shown in Table S11. Figures contain data ± SEM grouped from three independent biological replicates. Efficacy and potency values are summarized in Table S3.E) Summary of whole cell electrophysiological recordings from neurons in the ventral tegmental area (VTA) of acute rat brain slices, showing partial agonism of C6-Quino.

With the engagement of the allosteric sodium site of δOR, we were aiming for potent partial agonist activity in the G_i1_ protein signaling pathway and low efficacy in arrestin pathways. We found that, by varying the linker length, the potency and efficacy of bitopic ligands could be significantly changed. For example, **C5**- and **C6-Quino** displayed high potency, while **C7-Quino** showed diminished potency for G_i1_ (EC50 = 2.4 nM, 9.9 nM and 28 nM, respectively). While **C5-Quino** efficacy was close to a full agonist (E_max_ = 92 ± 1%), **C6**- and **C7-Quino** were partial agonists with reduced efficacies for G_i1_ (78 ± 1% and 77 ± 2% respectively) (Fig. 2A and 2B, Table S3). For β-arrestin1 recruitment, the potency of **C5-**, **C6-**, and **C7-Quino** was gradually reduced with increased linker length (EC_50_ = 28 nM, 190 nM, 600 nM; E_max_= 65 ± 2, 31 ± 2, 45 ± 3%, respectively). For β-arrestin2 recruitment, the potency and efficacy of **C5**, **C6-**, and **C7-Quino** was EC50 = 20 nM, 81 nM, 500 nM; E_max_= 85 ± 2, 45 ± 1, 75± 2%, respectively) (Figure S10, Table S3). The increase in efficacy of **C7-Quino** compared to **C6-Quino** is unclear, which again emphasizes the interactions with the sodium site could achieve efficacy modulation. While signaling profiles differed, the C5-C7 derivatives all maintained similar binding affinity for the δOR (Fig. 2C, Table S4).

Our assays also revealed that the potency and efficacy trends among the C5, C6, and C7 derivatives were consistent across the indole core (Fig. 2A and 2D, Table S3), indicating that these properties are influenced by factors beyond the orthosteric site, such as the linker length. Namely, bitopics with C3 (EC_50_ = 1.8 nM), C5 (2.8 nM) and C6 (5.9 nM) linkers were potent G_i1_ protein agonists, while ligands with the C7 linker showed 10-fold reduced G-protein potency (EC_50_ = 53 nM). Similarly, we saw a loss of efficacy across the series (81 ± 1%, 78 ± 2%, 47 ± 2% and 36 ± 2%, respectively) when compared to the reference DPDPE. Collectively, the potency and efficacy of guanidine derivatives showed a diminishing trend with the aliphatic linker chain length from C3, C5, C6 to C7 while maintaining similar binding affinity (Fig. 2A and 2C, Table S3 and S4).

Based on these data, our preferred lead partial agonist was **C6-Quino**, which has a higher δOR selectivity, reasonable potency, and lower intrinsic efficacy at both arrestin subtypes. To assess partial agonism within a physiologically native and endogenous system, we conducted whole cell electrophysiological recordings from neurons in the ventral tegmental area (VTA) in acute rat brain slices. Full δOR agonists like DPDPE and deltorphin have robust somatodendritic effects on VTA neurons^37^. We used voltage clamp experiments to measure changes in the holding current (*I*_holding_) induced by bath application of 10 μM **C6-Quino**. To establish a proper control, we performed similar experiments in separate brain slices from the same rats, measuring responses to 10 μM DPDPE. The distribution of responses to **C6-Quino** varied from responses to DPDPE, with the mean change in *I*_holding_ being close to 0 pA, suggesting that the partial agonism of **C6-Quino** maintains in vivo (Fig. 2E).

To better understand the signaling profile of our newly identified partial agonist, **C6-Quino** was next compared against other known δOR ligands across various chemical classes using the TRUPATH based G protein activation and arrestin recruitment assays^38^. Structurally and pharmacologically distinct ligands include peptides DPDPE, deltorphin II and Leu-Enkephalin (Leu-Enk); the diarylmethylpiperidines SNC80, SNC162, ARM390 and closely related ADL5959, and morphinan (±)-TAN67 (SB205607), in addition to **C5-Quino**. At G_i1_, **C6-Quino** showed the lowest efficacy among all ligands profiled. A similar pattern was seen at both arrestin subtypes as well, with the exception of (±)-TAN67 (Figure S11). While efficacies of (±)-TAN67 are comparable with **C6-Quino, C6-Quino** displays a much lower potency (EC_50_ = 31 nM, E_max_=45 ± 3%) in the β-arrestin2 pathway compared to (±)-TAN67 (EC_50_ = 1.1 nM, E_max_ =26 ± 3%) (Table S5, entry for SB205607). A similar tendency towards decreased intrinsic efficacy was seen at other Gα-subtypes for **C6-Quino** compared to other known δOR ligands though in this case both **C5-Quino** as well as **C6-Quino** efficacies were similar (Figure S11, Table S5). Overall, we conclude that bitopic engagement with the sodium site leads to reduced intrinsic efficacy at G-protein and even more at arrestin signaling pathways.

### CryoEM structures of δOR bound to bitopic ligands

**Figure 3:**
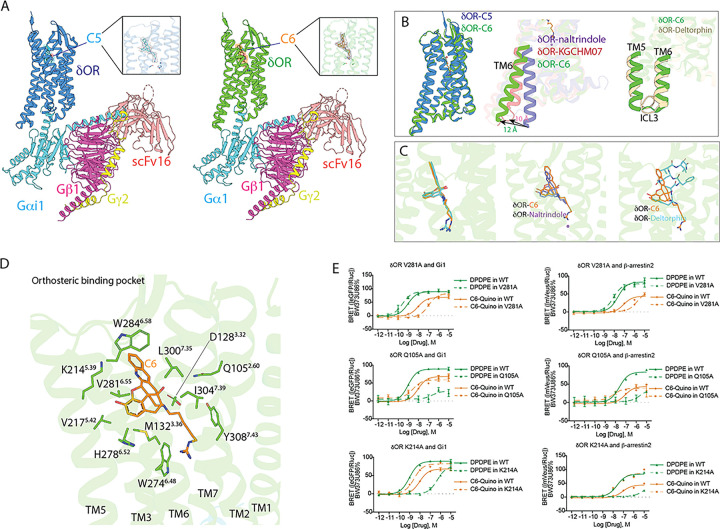
CryoEM structures of δOR bound to bitopics. A) Overall architecture of δOR-C6-Quino Gαi1Gβ1Gγ2 complex assembly. Density map of the ligand C6 is zoomed in. B) Comparison of C6-Quino bound δOR with previous inactive- and active-state δOR structure. δOR-naltrindole (PDB ID 4N6H), δOR-deltorphin (PDB ID 8F7S) C) Comparison of ligand binding pose between C6-Quino, naltrindole, and deltorphin. D) δOR-C6-Quino interactions in the orthosteric binding pocket. E) Residues with distinct effects on C6-Quino and DPDPE were characterized in BRET-G protein activation or arrestin recruitment assays. Potency and efficacy values are shown in Table S7.

To further confirm the interaction of the bitopic ligands with the δOR sodium site, we solved cryo-EM structures of **C5-** and **C6-Quino** (Fig. 3A) bound to δOR at a global 2.62 Å and 2.80 Å resolution, respectively (Figure S12 and S13, Table S6). The complex consists of δOR, **C5**- or **C6-Quino**, and Gαi1, Gβ1, and Gγ2 heterotrimers stabilized by ScFV16. It is worth pointing out that our active δOR-G protein structures do not include any thermostabilized mutations of the receptor as presented in previous active-like δOR structures with mutations in key motifs^39,40^, enabling more reliable analysis of the conformational changes responsible for receptor activation.

Both the δOR-**C5-Quino** and δOR-**C6-Quino** complex structures display a fully active-state and similar conformation in all the protein subunits with a root mean square deviation (r.m.s.d 0.6 Å), despite **C6-Quino** being a partial agonist (Fig. 3B). This is largely attributed to the binding of intracellular G protein heterotrimer that stabilizes the receptor conformation in this specific state. Both receptor conformations show a typical outward movement of the intracellular region of transmembrane helices VI (TM6) by 12 Å compared to the NTI bound δOR structure (Cα distance of S269^6.23^ compared to NTI bound inactive state, PDB: 4N6H) (Fig. 3B). This outward TM6 movement is a prominent feature of active state GPCR structures opening the intracellular site for G-protein binding. TM6 in **C6-Quino** structure also had additional outward movement compared to the 10 Å in the KGCHM07 agonist-bound δOR structure (PDB: 6PT3) (Fig. 3B). The additional outward TM6 movement is likely a result of G-protein binding in δOR-**C6-Quino**. Compared with the full agonist δOR-deltorphin-Gi1 protein structure, both TM5 and TM6 are in a similar position, although ICL3 appears to adopt different conformation (Fig. 3B). The density map is at high resolution, providing an unambiguous placement of the ICL3 in our structure. The displacement of ICL3 in the **C6-Quino** and deltorphin-bound δOR may contribute to the stability of ligand-specific ternary complex, because ICL3’s dynamic conformational equilibrium acts as an autoregulatory mechanism that impacts G-protein coupling to the receptor^41^.

As we hypothesized, the structures clearly show that both bitopic ligands occupy two pockets in δOR: an orthosteric ligand pocket and an allosteric sodium pocket (Fig. 3C). Both **C5-Quino** and **C6-Quino** bind similarly as NTI in the orthosteric site (Fig. 3C), which is expected since they were designed based on the NTI scaffold. Interestingly, **C6-Quino** and deltorphin barely share the binding site, with the exception of the phenol group present in the first tyrosine of deltorphin and morphinan portion of **C6-Quino**, each pointing to the TM5 (Fig. 3C). This difference in binding pose is notable because both **C6-Quino** and deltorphin are highly selective for δOR over other opioid receptor subtypes. One observation is that the quinoline ‘address group’ of **C6-Quino** forms strong hydrophobic interactions with the ECL3, particularly π – π interactions with W284^6.58^ (Fig. 3D), whereas deltorphin forms extensive interactions with ECL2^39^. Both are consistent with findings from structures of all four opioid receptors bound to their endogenous peptides, showing that the extracellular loops of opioid receptors act as filters for selectivity^40^.

**C5-Quino** and **C6-Quino** form conserved interactions with orthosteric pocket residues but display unique functional activity. The basic tertiary amine of **C6-Quino** interacts directly with the acidic residue D128^3.32^ through a salt bridge at 2.9 Å, a conserved interaction observed in other ligands bound to δOR (Fig. 3D).

To obtain insights into dynamics, 8 independent molecular dynamics (MD) simulations of 1000 ns each were performed for each complex. While both ligands remained bound in the pocket for all trajectories (Figure S14C and S14D), we observed different ratios of direct versus water-mediated-only interactions. (See Materials and Methods for additional details). **C5-Quino** maintained slightly closer contact to D128^3.32^ (**C5-Quino** 3.3 Å versus **C6-Quino** 3.5 Å on average) in the MD simulations; in both cases, direct interactions with D128^3.32^ were due to the protonated tertiary amine of the ligands, which can be accompanied by water-mediated interaction with D128^3.32^ due to the tertiary hydroxyl group near the protonated amine of the ligands. (Figure S14A, Figure S15A and S15D). By measuring autocorrelation C(t) of each water-mediated interaction, we also showed that water-mediated interaction at Y129^3.33^ was significantly more long-lived for **C5-Quino** (939 ± 52 ns) than **C6-Quino** (646 ± 34 ns). (Figure S15B-S15F). Otherwise, both **C5-** and **C6-Quino** showed substantial hydrophobic interactions at M132^3.36^, V217^5.42^, I277^6.51^, and W284^6.58^ (Figure S16).

We performed mutagenesis screening on residues that potentially interact with **C6-Quino** (Fig. 3E, Figure S17 and Figure S18). Whereas several mutations reduced the agonist activity of in **C6-Quino** G protein activation or arrestin recruitment, the mutations Q105^2.60^A and K214^5.39^A significantly increased the potency in Gi1 activation and β-arrestin2 recruitment (Fig. 3E, Figure S17, Table S7). This effect appears to be specific for **C6-Quino** as the two mutations almost abolished the activity and binding affinity of reference DPDPE (Fig. 3E, Figure S17, Table S7 and S8). The binding affinity of **C6-Quino** increases with a Q105^2.60^A mutation while it remains unchanged with the K214^5.39^A mutation (Figure S17, Table S8). The reason how Q105^2.60^A and K214^5.39^A are increasing signaling potency is difficult to determine but could be due to the removal of steric clash from the side chains after mutation, although molecular dynamics simulations found no direct contact at Q105^2.60^ with **C6-Quino** (Figure S15). In contrast, the mutation V281^6.55^A shows the opposite effect to Q105^2.60^A or K214^5.39^A, i.e., it leads to a loss (~ 11-fold) of activity for **C6-Quino**, but a 3-fold increase of activity of DPDPE. The binding affinity of DPDPE is increased in the V281^6.55^A mutation, suggesting the increase of activity could be attributed to better binding, potentially due to less steric clash (Figure S17, Table S8). The binding affinity of **C6-Quino** remains the same with the V281^6.55^A mutation which indicates that **C6-Quino** and DPDPE interact with this residue differently.

### Direct interactions with sodium site confer unique active-state like conformational changes

**Figure 4:**
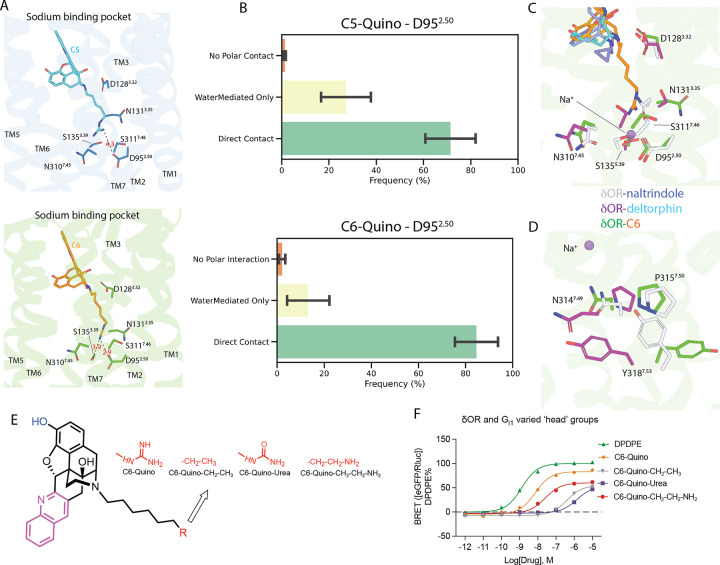
Interaction patterns in the sodium binding pocket. A) δOR-C6-Quino interactions in the sodium binding site. B) Statistics on Polar Contact between D95 and Ligands in MD Trajectories. Three coarse categories of polar contacts are presented in (**up**) for C5 and in (**bottom**) for C6; they are “No Polar Contact” (i.e. D95 does not participate in direct nor water-mediated interaction with the ligand), “WaterMediatedOnly” (i.e. there are water bridge(s) formed between ligand and residues in the sodium pocket without direct interaction with D95), and “Direct Interactions” (i.e. D95 is directly involved in the polar contact, which may or may not be supplemented by additional water bridge(s)). C) Conformational changes of sodium pockets residues between C6-Quino, naltrindole- and deltorphin-bound δOR. D The P-I-F motif, located at the bottom of the sodium site, undergoes unique conformational changes upon C6-Quino binding. E) Structures of quino derivatives with modified “warheads” F) Signaling of derivatives with different warheads demonstrates that polar interactions between ligand and allosteric site residues are crucial for ligand activity.

The extension of the guano group to the sodium site leads to a re-arrangement of the sodium-binding pocket residues (Fig. 4A), yet to different extents for C5 and C6 because of the linker length. The sodium site of δOR consists of several acidic and polar residues, D95^2.50^, N131^3.35^, S155^3.39^, N310^7.45^, and S311^7.46^, that coordinate the positively charged sodium ion in the inactive state δOR. Upon activation, these pocket residues undergo re-arrangements, leading to the disruption of the sodium-interacting networks and expulsion of the sodium ion. Specifically, **C6-Quino** forms a hydrogen bond with D95^2.50^ at 2.9 Å, and with S155^3.39^ at 3.9 Å, while **C6-Quino** that has a shorter linker forms weak interaction with the D95^2.50^ (4.3 Å) and no direct interactions with other sodium-site residues.

In MD simulations the guano group was predicted to almost always interact with D95^2.50^, with “direct contact” dominating over “water-mediated-only” interactions in both **C5-Quino** (70% versus 27%) and **C6-Quino** (84% versus 14%) (Fig. 4B). The “direct contact” in **C6-Quino** is slightly (not statistically significantly) more frequent than that in **C5-Quino**, though stronger direct contact is also supported by the slightly shorter average salt bridge distance to D95^2.50^ in **C6-Quino** (3.0 Å) than in **C5-Quino** (3.2 Å) (Figure S16). Interestingly, in **C5-Quino**, the direct salt bridge formed between the guanidine group of **C5-Quino** and D95^2.50^ was often supported by an additional water bridge with D95^2.50^ itself as well as water bridges with N310^7.45^ or S155^3.39^ (Fig. 4B, Figure S19). We also showed that while rapid exchange with the bulk solvent is common for those bridging waters in both ligands, **C5-Quino** had somewhat longer-lived water-mediated D95^2.50^ interaction than **C6-Quino** (correlation time 800 ± 71 ns versus 579 ± 79 ns) (Figure S19 B, E)

Mutation of sodium site residues D95^2.50^, N131^3.35^, S155^3.39^, and S311^7.46^ to alanine lead to non-functional receptors, making it difficult to study the effects of these residues on **C6-Quino** function (Figure S18, Table S10). The structural comparison between **C6-Quino** bound and NTI-bound δOR shows several significant conformational displacements of residues, including N131^3.35^, S155^3.39^, N310^7.45^, and S311^7.46^ which display 2–8 Å sidechain movement from inactive to active states (Fig. 4C). Interestingly, when compared with the deltorphin bound active-state δOR structure, N131^3.35^, N310^7.45^, and S311^7.46^, display further displacement in the C6 bound δOR structure. This is likely due to the disruption of charged interactions by the guanidine head of **C6-Quino**. As a direct effect of the altered arrangement in the sodium site, the NPxxY motif, adjacent to the sodium site, also displays unique conformations between **C6-Quino** and NTI or deltorphin bound δOR (Fig. 4D). However, this large displacement is not observed in another highly conserved DRY motif located in the intracellular end of TM3 that has been implicated in mediating the receptor activation and interactions with G proteins (Figure S20). These conserved sites have been implicated as important regulators in transducing the signal from the extracellular pocket to intracellular G protein coupling.

The idea that interactions with residues in the sodium binding pocket play roles in conformational dynamics and subsequent signaling transducer coupling was also corroborated by the differences caused solely by the length of the linker for guano compounds we presented earlier. To confirm this, we synthesized compounds with more neutral warheads like urea or polar warhead like amino, and with no warhead at all (Fig. 4E, Figure S6) to change the interaction patterns in the sodium site. As expected, we observed a drastic loss of potency and efficacy for C6-urea (EC_50_ = 1.4 μM and E_max_=53 ± 3% at G_i1_, arrestin not detectable), C6-quino-CH_2_CH_2_NH_2_ (EC_50_ = 25 nM and E_max_=60 ± 2% at G_i1_, EC_50_ = 180 nM and 170 nM with an E_max_ of 27 ± 2% and 46 ± 2% at β-arrestin1 and β-arrestin2), and all carbon C6-quino-CH_2_CH_3_ (EC_50_ = 390 nM and E_max_=55 ± 2% at G_i1_, EC_50_ = 2.1 and 1.0 μM with an E_max_ of 23% and 64 ± 2% at β-arrestin1 and β-arrestin2) compounds (Fig. 4F, see Figure S21 and Table S11 for arrestin data). However, the C6-urea also had a reduction in binding affinity, making it difficult to determine if reduced activity is due to sodium pocket interactions or binding affinity (Figure S22, Table S12).

The binding of these bitopic analogs was examined by molecular docking. Altering the guanidine moiety to carbamide in the context of the C6-quinoline-modified naltrindole scaffold yields a noticeable reduction in its interaction with the allosteric pocket in δOR. In the case of the guanidine structure, the positively charged guanidine effectively establishes a strong, end-on salt bridge interaction with the negatively charged D95^2.50^ residue^42^. Moreover, a robust hydrogen bond network is formed through guanidine, involving the basic carbonyl oxygen with S173^3.39^ and the amide hydrogen with S311^7.46^. In contrast, the neutral carbamide functionality only exhibits hydrogen bonding with D95^2.50^, alongside a hydrogen bond network similar to that formed by guanidine (Figure S23A and S23C).

For the C8-quinoline derivative featuring primary amine terminal group (Figure S23B), while a salt bridge interaction is present between the positively charged amine and D95^2.50^, this interaction is weaker than that of the guanidine warhead (Figure S23C, fewer ion-dipole interactions and hydrogen bonding in the case of the amine). The C8-amino-based scaffold is further stabilized by two hydrogen bonds, one with S173^3.39^ and another with N310^7.45^, contributing to its enhanced potency. Although it outperforms the C6-urea variant, it still falls short when compared to C6-guanidine quinoline-modified NTI-based scaffolds in terms of functional potency.

### C6-Quino displays δOR dependent anti-allodynia without adverse effects

**Figure 5:**
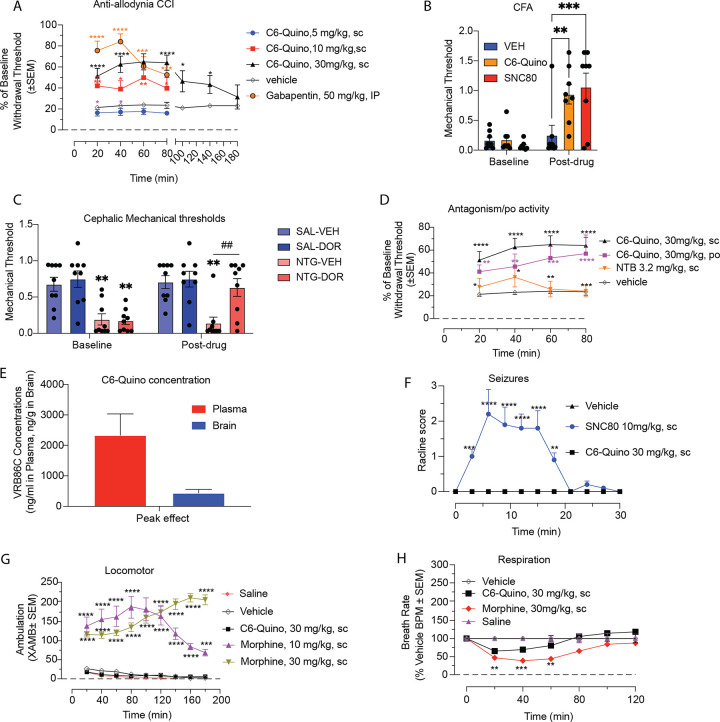
In vivo characterization of C6-Quino in chronic pain states. A) C6-Quino displays robust antiallodynic effect at both 10 mg/kg and 30 mg/kg dose similar to gabapentin at 50 mg/kg dose in the chronic constriction injury (CCI) model of neuropathic pain (Two-way RM ANOVA: treatment x time: F_(12,144)_=3.57, p=0.0001) and B) C6-Quino (30 mg/kg, sc) shows comparable anti-hyperalgesic effects to SNC80 (10mg/kg, sc) in the Complete Freund’s Adjuvant (CFA) model of inflammatory pain ((2-way ANOVA, p=0.0066 timeXtreatment interaction; Holm-Sidak post-hoc analysis **p=0.0025 comparing VEH-C6-Quino at Post-Drug time, ***p=0.006 comparing VEH-SNC80 at Post-Drug time). C) C6-Quino (30mg/kg, sc) completely reversed cephalic allodynia in the nitroglycerin model of chronic migraine (3-way ANOVA, p<0.05 timeXtreatmentXdrug interaction; Holm-Sidak post-hoc analysis **p<0.01 compared to SAL-VEH, ##p<0.01 compared to NTG/VEH). D) C6-Quino is effective in both po. and sc. regimes at 30 mg/kg dose and its effect can be antagonized with NTB in the chronic constriction injury (CCI) model (Two-way RM ANOVA: treatment x time: F_(12,129)_ =2.88, p=0.002). E) C6-quino shows reasonable brain and plasma exposure in pharmacokinetic assays in mice.). F) C6-Quino does not cause convulsions at 30 mg/kg dose unlike the full agonist, SNC80 at 10 mg/kg (sc.) (Two-way RM ANOVA: treatment x time: F_(20,231)_ =6.99, p<0.0001). G) C6-Quino, unlike morphine, does not produce hyperlocomotion (Two-way RM ANOVA: treatment x time: F_(32,567)_ =7.37, p<0.0001. H) Morphine causes significant decrease in respiration rate at 30 mg/kg. C6-Quino did not lead to significant respiratory depression at 30 mg/kg,sc when compared with vehicle. Respiratory rate. Mice were administered either vehicle (*n* = 12), morphine (30 mg/kg, sc; *n* = 12), or C6 Quino (30 mg/kg, sc; *n* = 12), and the breath rates was measured every 20 min for 120 min. Morphine administered sc caused reduction in the breath rate with respect to saline at 20 min (**p = 0.0029), 40 min (***p = 0.0004) and 60 min (**p = 0.0014) post drug administration. C6-Quino (30 mg/kg, sc) was not significantly different from vehicle control as determined by 2-way ANOVA followed by Dunnett’s multiple-comparison test.

To probe the possible utility of the partial agonist **C6-Quino** *in vivo*, we examined its effects in mice. First, we tested the anti-allodynic effects of **C6-Quino** in the chronic constriction injury (CCI) model of neuropathic pain. **C6-Quino** displayed dose-dependent inhibition of CCI-induced mechanical allodynia and a long lasting anti-allodynic effect when administered subcutaneously (sc.) at 30 mg/kg (Fig. 5A). **C6-Quino** was also effective at alleviating Complete Freund’s Adjuvant (CFA)-induced peripheral hyperalgesia and nitroglycerin (NTG)-induced cephalic allodynia at the same subcutaneous dose (Fig. 5B and 5C). The efficacy was comparable to the prototypic δOR agonist SNC80 administered at 10mg/kg, sc. **C6-Quino** was also orally active in the CCI assay after administration of a 30 mg/kg, p.o. dose, showing no significant difference in efficacy compared to sc. administration of the same dose (Fig. 5D). To assess the contribution of δOR to **C6-Quino** mediated analgesia, mice were pretreated for10 min with the δOR selective antagonist naltriben (NTB; 3.2 mg/kg sc.) prior to testing with **C6-Quino** (30 mg/kg sc.) in the CCI assay. Consistent with δOR being the major target of **C6-Quino**, NTB significantly blocked **C6-Quino** mediated anti-allodynia (Fig. 5D).

These results prompted us to evaluate the pharmacokinetics of **C6-Quino** after subcutaneous administration of a 30 mg/kg, dose. We measured concentrations of the intact drug in brain and plasma samples at peak effect (80 min), revealing plasma concentrations of ~ 1200 ng/ml and a brain-plasma ratio of 1:6. At the 80 min time point, > 100-fold higher drug concentrations were obtained in brain compared to the δOR G_i1_ EC_50_ concentration (Fig. 5E). In human liver microsomes, **C6-Quino** had a half-life > 2 hr, while sunitinib, a kinase inhibitor used as a reference, had a half-life of 27 min, **C6-Quino** also had a half-life > 8 hr in human plasma (Table S13). **C6-Quino**, carbamezapine and ritonavir had 88%, 65%, and 99.2% protein binding, respectively, in initial studies at human plasma, suggesting our current lead bitopic has > 5% free drug available for binding its target (Table S13).

Since δOR agonists are known to produce seizures^43^, we tested for the induction of pro-convulsant effects by **C6-Quino** at our highest analgesic dose (30 mg/kg, sc.), comparing it to the full δOR agonist SNC80 (10 mg/kg, sc.). While SNC80 produced robust seizures lasting 20 min, **C6-Quino** showed no signs of seizures in mice (Fig. 5F). Moreover, as seizures are attributed to a central δOR activity, we also administered C6-Quino intracerebroventricularly at a 100 nmol dose, with C6-Quino again showing no pro-convulsant activity. In contrast, SNC80 and (±)-TAN67 at the equivalent dose caused robust seizures, leading to euthanasia of all SNC80-treated mice at the end of 50 min (data not shown).

Pretreatment with **C6-Quino** (30 mg/kg, s.c.) produced neither hyper- nor hypolocomotion, while morphine at equianalgesic doses (10 or 30 mg/kg, s.c.) showed a significant increase in locomotion above baseline (Fig. 5G). In the continuous laboratory animal monitoring systems (CLAMS) assay as described before^20^, morphine showed respiratory depression while **C6-Quino** showed no decrease in breath rates at 30 mg/kg, sc. dose, although a non-significant decrease in breath rates was observed shortly after administration (Fig. 5H).

In summary, our approach to target an allosteric sodium site with a charged polar head group in bitopic ligands yielded *in vivo* analgesic efficacy without typically observed opioid adverse effects. Most importantly, for a ligand showing δOR agonism, **C6-Quino** did not cause seizures when administered either peripherally or centrally.

## DISCUSSION

In this study focusing on δOR, we employed a structure-based approach to design partial agonists by simultaneously targeting the orthosteric site and the sodium binding pocket. This bitopic approach allows us to modulate signaling efficacy at G-proteins as well as β-arrestins, to developing safer pharmacological options for the treatment of chronic pain.

Chronic pain poses a significant and complex health challenge, necessitating the development of safe and effective analgesics that avoid the adverse effects and addictive potential of conventional opioids. While μOR agonists have demonstrated effectiveness for acute pain, they are accompanied by severe side effects and abuse liability. In contrast, δOR agonists have shown promise in rodent models of chronic pain and generally lack the undesired effects of μOR agonists^3^, including respiratory depression, addiction liability, or constipation. Therefore, they hold significant promise as targets for pain-relieving therapies in the treatment of neuropathic and inflammatory pain, conditions that currently lack effective treatment options^44^. The major limitation to δOR agonist development in the past is that selective ligands such as SNC80 and SNC162 have produced convulsions, rendering them unsuitable for clinical use^3^.

Recently two competing theories have been proposed to explain why certain drugs have beneficial pharmacological profiles: biased signaling and low efficacy signaling^45–48^. Biased signaling, where a ligand can activate specific signaling pathways or cellular responses through a receptor while not fully activating all possible pathways has been proposed as a method to achieve safe therapeutics by fine-tuning of the pharmacological effects. However, achieving biased signaling poses a challenge as it requires a deep understanding of the receptor’s molecular mechanisms and the ligand's interaction with it. Low efficacy, on the other hand, refers to the partial agonisťs reduced ability to activate a receptor compared to a full agonist. Developing partial agonists with the right balance of efficacy necessitates finding a pharmacological “sweet spot”, where the ligand provides therapeutic benefits while avoiding overstimulation or inadequate activation of the receptor.

In line with this emerging need, our investigation focused on the transition from full to partial agonism. Intriguingly, as we engaged the sodium binding site, we observed such a transition between **C5-Quino** and **C6-Quino**. This difference in efficacy was even more pronounced in arrestin activity than in G-protein signaling. To better understand this difference in efficacy we combined structural and pharmacological approaches to confirm that interactions with the sodium site play important roles in ligand efficacy modulation.

How receptor efficacy is modulated has been previously unclear. A recent study uncovered the mechanism responsible for xanomeline's efficacy-driven selectivity among closely related muscarinic acetylcholine receptors (mAChRs)^49^. Xanomeline binds similarly to the inactive states of all five mAChR subtypes but differently to their active states, due to steric clashes of its tail with the second extracellular loop. Changes in efficacy are driven by the contraction of the binding pockets upon activation, a mechanism that could apply to other G-protein-coupled receptors (GPCRs) where binding pockets change size during activation. These findings indicate that small structural changes, like adding a few atoms to a drug molecule, can alter efficacy. These changes can be explained through classical thermodynamics of binding at the orthosteric site, without considering factors like binding kinetics, receptor internalization, or receptor oligomerization.

In summary, **C6-Quino** displayed potent analgesic effects without typical δOR adverse effects. Its unique properties, including the absence of seizures, locomotor impairment and reduced respiratory depression, highlight its potential as a candidate for chronic pain treatment. These findings also suggest that targeting the sodium site offers a new approach towards modulating signaling activity, thereby opening up new opportunities for the development of analgesics with enhanced safety profiles and efficacy in treating chronic pain, particularly in neuropathic and inflammatory conditions where current treatment options are limited. Further research and clinical trials will help validate the potential of δOR partial agonists as safer therapeutic agents for chronic pain. Moreover, the approach presented here serves as a versatile strategy for enhancing the signaling characteristics of other Class A GPCRs, making it a valuable tool for optimizing their performance.
